# Usability of a virtual reality environment simulating an automated teller machine for assessing and training persons with acquired brain injury

**DOI:** 10.1186/1743-0003-7-19

**Published:** 2010-04-30

**Authors:** Kenneth NK Fong, Kathy YY Chow, Bianca CH Chan, Kino CK Lam, Jeff CK Lee, Teresa HY Li, Elaine WH Yan, Asta TY Wong

**Affiliations:** 1 Department of Rehabilitation Sciences, The Hong Kong Polytechnic University, Hong Kong; 2 Occupational Therapy Department, Kowloon Hospital, Hong Kong

## Abstract

**Objective:**

This study aimed to examine the usability of a newly designed virtual reality (VR) environment simulating the operation of an automated teller machine (ATM) for assessment and training.

**Design:**

Part I involved evaluation of the sensitivity and specificity of a non-immersive VR program simulating an ATM (VR-ATM). Part II consisted of a clinical trial providing baseline and post-intervention outcome assessments.

**Setting:**

A rehabilitation hospital and university-based teaching facilities were used as the setting.

**Participants:**

A total of 24 persons in the community with acquired brain injury (ABI) - 14 in Part I and 10 in Part II - made up the participants in the study.

**Interventions:**

In Part I, participants were randomized to receive instruction in either an "early" or a "late" VR-ATM program and were assessed using both the VR program and a real ATM. In Part II, participants were assigned in matched pairs to either VR training or computer-assisted instruction (CAI) teaching programs for six 1-hour sessions over a three-week period.

**Outcome Measures:**

Two behavioral checklists based on activity analysis of cash withdrawals and money transfers using a real ATM were used to measure average reaction time, percentage of incorrect responses, level of cues required, and time spent as generated by the VR system; also used was the Neurobehavioral Cognitive Status Examination.

**Results:**

The sensitivity of the VR-ATM was 100% for cash withdrawals and 83.3% for money transfers, and the specificity was 83% and 75%, respectively. For cash withdrawals, the average reaction time of the VR group was significantly shorter than that of the CAI group (p = 0.021). We found no significant differences in average reaction time or accuracy between groups for money transfers, although we did note positive improvement for the VR-ATM group.

**Conclusion:**

We found the VR-ATM to be usable as a valid assessment and training tool for relearning the use of ATMs prior to real-life practice in persons with ABI.

## Introduction

Operating an automated teller machine (ATM) is one of the most common tasks involving community-living skills that a person might undertake. ATMs are computerized telecommunication devices that provide the customers of financial institutions access to making financial transactions in a public space without the need for a human clerk or bank teller. Using an ATM, customers can access their bank accounts to make cash withdrawals, transfer money, and check the balance of their accounts, as well as pay electronic bills. But although ATMs are widely used and very convenient, it has been shown that older persons and users with disabilities face difficulties with their operation [1,2]. A study of the elderly conducted in Japan showed that these difficulties may result not only from sensory problems and physical characteristics but also from cognitive changes as a result of aging, all of which make it difficult for elderly persons to understand ATM operations and the meaning of the ATM display [2]. Using an ATM simulator, the researchers showed that common problems affecting the elderly in their use of ATMs were 1) long response times, 2) difficulties collecting information in a short time, 3) excess response to voice messages, 4) recurrence of the same errors, 5) difficulties understanding the operational tasks, and 6) the influence of social pressure.

In addition, persons with acquired brain injuries (ABI) have different levels of cognitive function that can affect their ability to perform basic self-care and participate in the community [3]. They may also lack the ability to operate an ATM either because of cognitive deficits like memory difficulty, poor problem-solving, or slow motor and information processing speed, or a lack of confidence from insufficient practice. With a real ATM, people can lose their ATM card after three mistakes during password key-in. Also, if their response is delayed more than 30 seconds, their card will be rejected and they must start over again. The frustration produced by the time limit and the demand for accuracy, as well as anxiety stemming from the possibility of long lines developing behind them in a public area, also makes it more likely such individuals will avoid this community activity. But although studies have been undertaken of ATM use by the elderly [2], there has yet to be a study investigating the use of ATMs by other populations, including people with disabilities.

Virtual reality (VR) is a technology that allows people to view, navigate, or interact with a simulated three-dimensional world in real time. It is a computer-generated environment that creates an opportunity for individuals to engage in activities similar to the reality [4-6]. These environments are usually three-dimensional and can be classified into two types which are immersive and non-immersive depending on the levels of "virtual presence" [5]. Different VR systems come with different kinds of interactivity according to different immersion levels. In an immersive environment, individuals will have strong "sense of presence" and are able to view themselves or an avatar in the scene on the screen using head-mounted display or cave system, however, in a non-immersive system, individuals only interact with the environment displayed on the computer screen with or without interface or haptic devices [5]. VR offers the chance for intensive repetition of meaningful tasks with augmented feedbacks for rehabilitation in a manner that can be more interesting than conventional therapy [6]. It poses no threat to or physical limitations upon participants in the simulated environment, and it can easily be modified to change levels of difficulty, which may not be possible in the real world [7]. It also has advantages over normal computer-based rehabilitation programs in that it can address real-time aspects of information processing and enhance dynamic interaction [5]. Previous studies have empirically substantiated the usefulness of VR in the relearning of domain-specific functional tasks, such as cooking, route finding, and cash management, in rehabilitating clients with cognitive impairments such as brain injury [3,7-11]. But no study so far has attempted to create a virtual ATM environment for the training and studying of persons with disabilities or the elderly in need of developing the necessary skills for ATM use. The potential benefits of such an environment led to our project to design and evaluate a virtual ATM in which persons with ABI could practice the necessary skills during their daily rehabilitation sessions [12].

In this study, we sought to answer two questions: 1) What would be the value of using a newly designed virtual reality ATM program (VR-ATM) in predicting the success or failure of persons with ABI when using real ATMs? and 2) would the VR-ATM be an effective training media compared with the conventional training approach using computer-assisted instruction (CAI) for improving the performance of persons with ABI in cash withdrawals and money transfers at ATMs? The study was divided into two parts: the first investigated the predictive validity of using the VR-ATM as a tool to assess performance on real ATMs in persons with ABI, while the second compared the effects of using the VR-ATM program with those of using CAI when training persons with ABI to operate ATMs for cash withdrawals and money transfers. We believed that task-specific training through the use of VR could enhance representative functioning in problem-solving in clients with ABI, which might then be incorporated into the various stages of problem-solving skills training and then transferred to problem-solving performance in real life [13].

## Methods

### Participants

A total of 24 participants with ABI volunteered to participate in the study. To be included, participants had to 1) be aged between 18 and 65, 2) have the ability to use at least one hand to operate the touch monitor, 3) attain a score of 22 or above on the Chinese Mini-Mental State Examination (MMSE) [14], 4) be capable of going outdoors independently, and 5) not have used an ATM since the injury. Participants were excluded if they had severe dysphasia (either expressive or comprehensive), which restricts communication, or significant impairment in visual acuity caused by cataracts, diabetic retinopathy, glaucoma, or hemianopia. All participants were diagnosed with ABI according to their medical records, which is an injury to the brain that has occurred after birth and that is not hereditary, congenital, degenerative, or been induced by birth trauma [15]. For the first part of the study, we recruited 14 outpatients with ABI by convenience sampling in the occupational therapy department of a rehabilitation hospital in Hong Kong. The patients ranged in age from 18 to 59 years (mean = 43; SD = 10.7). Nine were diagnosed with stroke (6 hemorrhagic, 3 ischemic), and four had sustained head injuries (1 closed, 4 open). For the second part, we recruited 10 participants by convenience sampling from a self-help community organization in Hong Kong. These participants had experienced the onset of ABI more than one year previously and had completed the outpatient rehabilitation phase of their treatment, thus minimizing the chance of spontaneous recovery. Their ages ranged from 44 to 63 years (mean = 52.6; SD = 6.2). Nine were diagnosed with stroke (6 hemorrhagic, 3 ischemic), and one had suffered a brain tumor. Table 1 shows the cognitive performance of the participants in both parts of the study. Written and informed consent was obtained from all participants before study enrollment. The study was performed in accordance with the principles of the Declaration of Helsinki, and was reviewed and approved by the institutional review board of the Hong Kong Polytechnic University (Ref.: HSEARS20061222001).

**Table 1:** Characteristics of all participants

Characteristics	Part I (n = 14)	Part II (n = 10)
*Gender, n(%)*		
Male	11 (78.6)	6 (60.0)
Female	3 (21.4)	4 (40.0)
*Age, mean ± SD*	43.0 ± 10.7	52.6 ± 6.2
*Years of education, mean ± SD*	10.6 ± 2.7	8.0 ± 3.7
*Education, n(%)*		
No formal education	0 (0)	1 (10.0)
Primary	1 (7.1)	3 (30.0)
Lower secondary	5 (35.7)	3 (30.0)
Upper secondary	6 (42.9)	3 (30.0)
Tertiary	2 (14.3)	0 (0)
*Type of ABI, n (%)*		
Intracerebral hemorrhage	6 (42.8)	6 (60.0)
Ischemic stroke	3 (21.4)	3 (30.0)
Open head injury	4 (28.6)	0 (0)
Closed head injury	1 (7.0)	0 (0)
Brain tumor	0 (0)	1 (10.0)
*MMSE, mean ± SD*	27.6 ± 2.7	26.1 ± 2.3

Note: MMSE - Mini-mental State Examination

### Instrumentation

**Figure 1:**
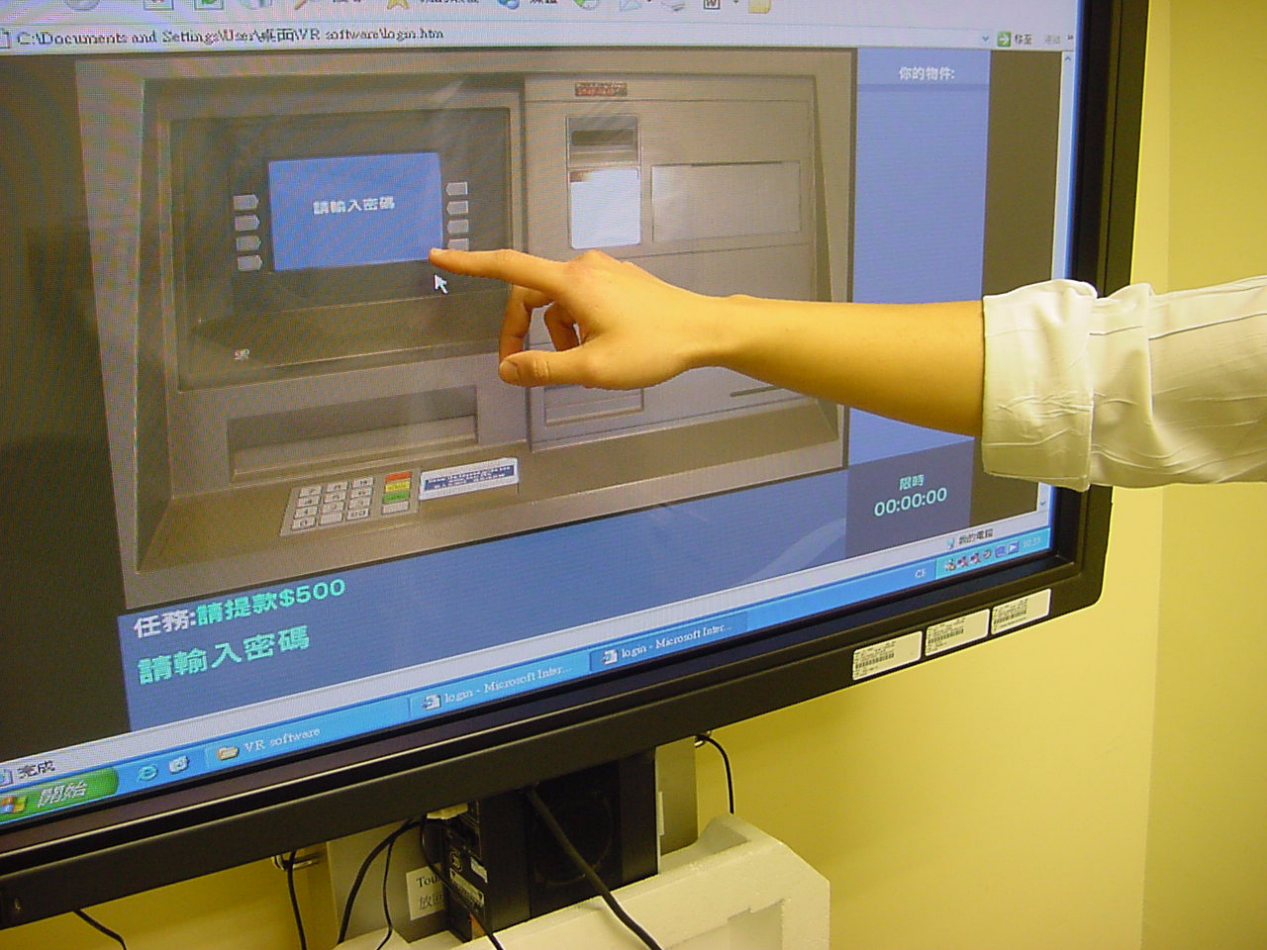
**Non-immersive virtual reality ATM program (VR-ATM)**.

Figure 1 shows the non-immersive virtual reality ATM program (VR-ATM) developed at the occupational therapy department of Kowloon Hospital, Hong Kong, in 2005 [12]. It is operated online through a web-based system. The system comprises a desktop computer connected to the internet with a 27" touch monitor that closely represents the anticipated actual dimensions of a real ATM. The objective in developing the VR-ATM was not to employ sophisticated technology but to construct a virtual environment that could be manipulated with assessment and training parameters designed to enhance task performance, as well as be accessible everywhere through the internet. The VR-ATM includes three common tasks that can be conducted at ATMs: cash withdrawals, money transfers, and electronic payments. The program in the system consisted of two modes: assessment and training. Augmented feedback to the individual is enhanced by visual and auditory feedbacks to the results of actions. In each task, a cueing system, consisting of five levels of cues, was programmed to respond to the actions of the participants and assist them with task completion. When a delay in response was detected, cues were given in 15-second intervals. The cues were sequenced as follows according to the level of assistance provided by the system: 1) whenever the user performed incorrectly, the program provided a reminder signal and the user had the opportunity to retry; 2) if the user again answered incorrectly, a cue in the form of a flashing object could be seen over the correct button to press; 3) if the user still did not answer correctly, a verbal cue in the form of a voice was provided; and 4) if the user still could not complete the task, a bright arrow cue pointing to the correct button was provided. If the response was still incorrect or not completed, the computer performed the task and a final score was generated that took account of the level of cues given. The advantage of this algorithm in terms of feedback to the user is that it provides the necessary reinforcement or prompting for the initiation of activity, the sequence of steps, and the correctness of actions [11]. An assessment report was produced after each attempt that included outcomes such as average reaction time, percentage of incorrect responses, level of cues required, and time spent on the task. The training mode was also divided into three levels of difficulty. Level 1 involved only the simplest task of cash withdrawals; Level 2 involved any two tasks out of cash withdrawals, money transfers, or electronic payment; and Level 3 involved all three tasks.

### Procedure

#### Part I

The first part of the study involved a one-time instruction session in use of both the VR-ATM and the real ATM by an occupational therapist. Participants were first randomly allocated by sealed envelopes to either an "early" or a "late" VR-ATM program. The early group received instruction in the VR-ATM program, immediately followed by practice with the real ATM. Participants in the late group were exposed to the real ATM first, immediately followed by the VR-ATM. All participants performed the same tasks - cash withdrawals and money transfers - with the VR-ATM or the real ATM in a rehabilitation hospital. For the real ATM practice, the therapist issued the participant an ATM card, a password, and the account number of the target account for the money transfers. The assessment criteria were based simply on a dichotomous scale - success or failure on a behavioral checklist in using the ATM (see Additional file 1).

#### Part II

The second part of the study used a pre-test and post-test quasi-experimental design that involved two training groups - the experimental group and a conventional group. Participants with ABI were assigned by matched pairs into either the group using the experimental VR-ATM program or the group using the conventional CAI program [16], and were taught separately by two trained occupational therapy students in a university-based teaching laboratory. They were matched in pairs as closely as possible in terms of age, gender, educational level, and baseline cognitive performance as assessed by the MMSE. Both programs consisted of six equivalent one-hour training sessions, two sessions per week for three weeks, with each session involving the same content and structure for instructing participants in basic ATM skills for withdrawing cash (Level 1) and transferring money (Level 2); both themes were included in different sessions using either VR or CAI, respectively.

The VR-ATM program allowed participants to manipulate objects such as a debit card, receipt, or cash in the virtual world using a touch-screen person-machine interface. They practiced and learned to use the VR-ATM through tasks such as inserting a debit card, withdrawing cash, and entering a password. The amount of cash to be withdrawn or transferred varied randomly as set by the system, although the amount used in the intervention was not the same as that used in the pre- and post-testing stages. The trainer also taught the participants cash management and simple calculation during money transactions with the VR-ATM.

The CAI program adopted the same structure and content as that of the VR-ATM program. It combined multimedia tutorials (using PowerPoint) with feedback and verbal reinforcement. The only difference was that the participants in the CAI group received tutorial sessions, demonstrations, and verbal feedback from the trainer with the use of PowerPoint rather than from a virtual environment.

### Outcome measures

In Part I, the success or failure of participants in using either the VR-ATM or the real ATM was recorded and compared for each step. Two behavioral checklists were developed based on activity analysis, a 14-item one for cash withdrawals and a 17-item one for money transfers, to assess the participants' performance in using ATMs (see Additional file 1) [17]. The checklists consisted of items that broke down the procedures of basic ATM operations, cash withdrawals, and money transfers into smaller steps. We used a dichotomous scale of 0 and 1 based on a pass or fail principle to score performance. We also evaluated their cognitive performance using the Neurobehavioral Cognitive Status Examination (Cognistat) [18] before practice with the ATMs. The Cognistat was developed in 1983 by the Northern California Neurobehavioral Group, Inc. which used an ability model of brain function emphasizing different areas of cognitive function. In this study, the sub-scores of two general areas, attention and orientation, and five major ability areas namely language, constructions, memory, calculations and reasoning, were tested [19]. There was no blinding of group assignment by the assessor.

In Part II, the VR-ATM system produced reports of major outcomes before and after training, which included the average reaction time, percentage of incorrect responses, number of cues needed, and time spent. The pre- and post-assessments also included cognitive evaluation using Cognistat. An occupational therapy student was specially trained to oversee all assessments in Part II.

### Data analysis

The baseline and demographic data of all participants were assessed by descriptive statistics. In Part I, we compared the performance of participants in the two groups with the VR-ATM and the real ATM based on four possible outcomes - true or false positives and false or true negatives - in a 2 × 2 arena, as shown in Table 2[20]. Sensitivity was calculated according to the power of the VR-ATM to obtain a true positive result; that is, a/(a+c), where a was the true positive and c was the false negative. This value is the portion of participants who failed both when using the VR-ATM and the real ATM. Another value, a/a+b, was calculated that reflected the positive predictive value, that is, the VR-ATM's ability to detect problems in those participants who failed when operating the real ATM. Specificity was calculated according to the power of the VR-ATM to obtain a true negative result, which was calculated as d/(b+d), where b was a false positive and d was the true negative. This value represented the proportion of participants who succeeded at operating the VR-ATM but who would fail in real practice. A negative predictive value of d/c+d indicated those who succeeded in operating the VR-ATM who would also succeed at using the real ATM. Hence, if the VR-ATM had a high negative predictive value, everyone who succeeded in operating the VR-ATM would have no difficulty using a real ATM. Part II of the study used non-parametric statistical methods owing to the small sample size. The Mann-Whitney Test was used to detect differences in both baseline and post-training evaluation between groups. The level of significance was set at p ≦ 0.05.

**Table 2:** Predictive values (by case) of VR-ATM and real ATM in outpatients with ABI (n = 14)

Observation (by case)	Real ATM			
	
	Cash withdrawals		Money transfers	
	
	Failure (n)	Success (n)	Failure (n)	Success (n)
VR-ATM	2	2	5	2
Failure	(a:true positive)	(b:false positive)	(a:true positive)	(b:false positive)
VR-ATM	0	10	1	6
Success	(c:false negative)	(d:true negative)	(c:false negative)	(d:true negative)

Note: Figures represent the number of participants

## Results

### Part I

For those who succeeded at operating the real ATM, average reaction time was 15.5 seconds (range was 12 to 19 seconds) with the level of cues ranging from 1 to 2 with the VR-ATM. For those who failed with the real ATM, average reaction time was 26.5 seconds (range was 23 to 30 seconds) with the level of cues ranging from 2 to 3 with the VR-ATM.

The sensitivity of the VR-ATM was 100% for cash withdrawals and 83.3% for money transfers. This meant that if the participant lacked sufficient ability to operate the real ATM, the VR-ATM would accurately reflect the deficits. This result reflects a high probability that the VR-ATM would detect problems in users who would fail at using a real ATM. The specificity of the VR-ATM was 83% and 75% for cash withdrawals and money transfers, respectively. This result reflects satisfactory specific values but some probability that the VR-ATM would detect problems in clients who would actually succeed in using a real ATM. The VR-ATM had an acceptable positive predictive value of 50%, meaning that it estimated that half the users who had problems operating the VR-ATM would fail when using a real ATM. The VR-ATM, however, had a high negative predictive value of 100%; in other words, every participant who succeeded in operating the VR-ATM would have no difficulty using a real ATM.

### Part II

The results of the Mann-Whitney test indicated no significant differences in cognitive performance between participants in the VR-ATM and CAI groups as assessed by the Cognistat (p = 0.288 - 0.911), and in baselines as assessed by the VR-ATM (p = 0.753 - 0.834) (Table 3). Table 4 summarizes the outcome measures comparing participants' performance in both groups after training as assessed by the VR-ATM. The results of the Mann-Whitney test also indicated no significant differences in baseline measures prior to the intervention in terms of age, years of education, and the Cognistat, outcomes of average reaction times, and correct percentage scores on the VR-ATM (Table 3).

**Table 3:** Results of Mann-Whitney Test in baseline comparison between groups

	VR-ATM Group (n = 5)	CAI Group (n = 5)	Z	p
*Age*	53.2 ± 7.5	52.0 ± 5.5	-0.315	0.841
*Years of education*	7.0 ± 4.3	9.0 ± 2.5	-0.764	0.548
*Gender, no. of M/F*	(3/2)	(3/2)		
*Cognistat*				
Orientation	10.4 ± 2.6	11.2 ± 1.79	-0.643	0.521
Attention	7.8 ± 0.5	7.6 ± 0.9	-0.149	0.881
Comprehension	5.4 ± 1.3	5.2 ± 1.3	-0.516	0.606
Repetition	8.8 ± 2.2	7.6 ± 3.2	-0.532	0.595
Naming	6.0 ± 1.4	7.6 ± 4.4	-0.324	0.746
Constructional ability	3.8 ± 1.6	4.2 ± 1.5	-0.328	0.743
Memory	8.4 ± 2.3	8.0 ± 3.2	-0.212	0.832
Calculation	3.0 ± 1.2	3.2 ± 0.8	-0.111	0.911
Reasoning	5.8 ± 2.5	6.6 ± 0.9	-1.063	0.288
Judgment	4.0 ± 0.7	4.6 ± 0.9	-0.437	0.662
*Cash withdrawals*				
Average reaction time	15.8 ± 8.5	16.6 ± 6.7	-0.314	0.753
Correct percentage score	87.7 ± 11.3	85.1 ± 11.6	-0.313	0.754
*Money transfers*				
Average reaction time	21.0 ± 4.0	24.2 ± 9.6	-0.424	0.671
Correct percentage score	78.2 ± 5.2	72.5 ± 13.1	-0.210	0.834

Mean ± SD; * denotes significance at p ≦ 0.05

Note: Cognistat - Neurobehavioral Cognitive Status Examination

Table 4 also shows the results of between-group comparisons in post-training VR-ATM outcomes. For cash withdrawals, the experimental group had a significantly shorter average reaction time than the CAI group (p = 0.021). The accuracy of the VR-ATM group was also significantly higher than that of the CAI group in cash withdrawals (p = 0.043). For money transfers, no significant difference was found in the average reaction times of the two groups after training (p = 0.173), although the reaction time for the VR-ATM group did show positive improvement (mean = 10.6) and was shorter than that of the CAI group (mean = 16.6). We also found no statistically significant difference in the post-test correct percentage scores between the VR-ATM and CAI groups, although the improvement in accuracy for the VR-ATM group almost allowed the comparison to meet the 0.05 cut-off point for significance (p = 0.059) (Table 4).

**Table 4:** Results of Mann-Whitney Test in post-training comparison between groups

		VR-ATM Group (n = 5)	CAI Group (n = 5)	Z	p
*Cash withdrawals*	Average reaction time	5.6 ± 2.1	15.2 ± 6.5	-2.312	0.021*
	Correct percentage score	98.9 ± 2.5	89.4 ± 8.4	-2.019	0.043*
*Money transfers*	Average reaction time	10.6 ± 5.7	16.6 ± 7.2	-1.362	0.173
	Correct percentage score	93.2 ± 5.8	82.7 ± 9.5	-1.886	0.059
*Cognistat*	Orientation	11.6 (0.9)	11.8 (0.5)	-0.149	0.881
	Attention	8.0 (0.0)	7.4 (0.9)	-1.491	0.136
	Comprehension	5.6 (0.9)	5.6 (0.9)	0.000	1.000
	Repetition	10.0 (1.0)	8.8 (2.6)	-0.435	0.663
	Naming	6.4 (1.5)	6.6 (1.7)	-0.217	0.828
	Constructional Ability	5.6 (0.9)	4.4 (1.5)	-1.423	0.155
	Memory	10.2 (0.8)	8.8 (2.8)	-0.212	0.832
	Calculation	3.2 (1.3)	3.2 (1.3)	0.000	1.000
	Judgment	6.8 (0.8)	6.8 (1.3)	-0.767	0.443
	Reasoning	4.2 (2.1)	5.2 (0.8)	-0.108	0.914

Mean ± SD; * denotes significance at p ≦ 0.05

Note: Cognistat - Neurobehavioral Cognitive Status Examination

## Discussion

Our study shows that the VR-ATM can be used both as a reliable assessment tool to assess performance in using ATMs and as a useful program for training clients with ABI in ATM user skills. Given the popularity of internet use in Hong Kong, we provided a web-based system for VR training in ATM skills that would allow persons with disabilities or older persons to practice in locations outside the treatment center, including at home, thereby increasing the accessibility, duration, and frequency of practice with or without help from caregivers while saving the time it would take to travel to the treatment center.

Part I of the study found the VR-ATM program to be a valid and highly sensitive screening tool for assessing the ATM user skills of patients with ABI, and showed that the VR-ATM is an effective assessment tool that can identify clients who would have problems with real ATM operation. The sensitivity of the VR-ATM was 100% for cash withdrawals and 83.3% for money transfers, which reflects the high probability of detecting problems with VR-ATM use in clients who would be likely to fail when using real ATMs. In view of the good result for the negative predictive value - that is, predicting participant success when using the VR-ATM - it is likely that 100% of those who succeeded in using the VR-ATM could also succeed at operating a real ATM. If participants failed at operating the VR-ATM, they had a 50% chance of succeeding when using the real ATM, as shown by the positive predictive value. This means that if a person lacked sufficient ability to operate a real ATM, the VR-ATM would accurately reflect this problem. The VR-ATM can therefore be used as an assessment tool to screen clients with ABI who are likely to have difficulty using real ATMs.

But the possibility of failure when using real ATMs, as reflected by the 50% positive predictive value, could also be attributed to environmental distractions in the public area and the threat of losing the ATM card after three mistaken password key-ins. We recommend that people with ABI do not try using ATMs in a public area until they are able to successfully operate the VR-ATM. All persons face a high level of environmental distractions, disturbances from unreasonable complaints, and social pressure created by the line behind them, all of which can affect concentration and frustration but may impact those with ABI more significantly. We found that the VR-ATM was not highly specific for money transfers (75%); that is, participants who had no difficulty with a real ATM could still fail when operating the VR-ATM, possibly owing to the complexity of or unfamiliarity with the system, as also reflected in reliability studies of virtual environments [7]. In addition, we found that participants with slow motor speed had difficulties managing touch screen monitors, which are more sensitive in response than the buttons of a real ATM. This result was consistent with our previous finding that individuals with brain injuries showed a clear slowing in reaction time and a tendency to trade off time for accuracy [21]. Although it was noted that all participants were cognitively intact as reported in the MMSE results (Table 1), the test did not detect slowness of information processing or impaired executive functioning which are common general cognitive impairments after acquired brain injury.

Success in real ATM practice can thus be predicted by the average reaction time and level of cues used in the VR-ATM program. In reality, participants with an average reaction time exceeding 30 seconds in any step would fail when using a real ATM. Participants who failed in operating the VR-ATM usually needed an average of 26.5 seconds (ranging from 23 to 30 seconds) in average reaction time with more than two levels of cues. Those who passed the real ATM test showed a mean of 15.5 seconds (ranging from 12 to 19 seconds) and a range between one and two cue levels in the VR-ATM. It is likely that people with ABI fail at real ATM operations because of their slow response time, which cannot be traded off since a real ATM is programmed to allow every person a maximum of 30 seconds to respond at every step and only three chances to make an error in password key-in.

In Part II, our baselines showed no significant differences between the two groups, implying that the method of assigning participants to either group by matched pairs was successful in equalizing both. Thus, the results of the study support use of the VR-ATM to train people with ABI as a better approach than using conventional CAI in improving speed and accuracy in making cash withdrawals. In the performance of money transfers, the difference between groups was close to significant, but this advantage was lost in the post-training results. Failure to achieve a statistically significant difference in this finding may be related to the complex steps involved in making a money transfer, since the cash withdrawal task was simple and had fewer steps compared with the money transfer task.

VR technology can serve as a program for repetitive practice in a simulated and modifiable environment that poses no threat to participants and places no physical limitations upon them. Repetitive practice is essential to effective therapy, and the VR approach provides an objective, accurate measurement of patient responses in a series of repetitive tasks and a more economical training program requiring less than one-on-one contact with a therapist [11]. Once clients with ABI are referred to outpatient rehabilitation and their problems and difficulties using ATMs have been identified, repetitive skills training with the VR-ATM could improve reaction time, accuracy, and the client's confidence. If the client was successful, he or she could proceed to real ATM operation under supervision by staff or a family member. In the future, more virtual community living skills programs beyond the ATM could be developed to increase training opportunities for clients with ABI or other cognitive disabilities and to facilitate community reintegration.

This study does have several limitations. The sample size in both parts of the study was small. During the real ATM practice in Part I, the money amount used for money transfers was only HK$10, which was too small a value to induce anxiety comparable to a real-life situation. A follow-up measurement in Part II of the study would also be necessary to determine whether the skills gained from using the VR-ATM are maintained over time. Furthermore, the long-term benefits of the VR-ATM for learning to reuse ATMs prior to real-life practice in clients with ABI remain unproven. Although the VR-ATM can be used as a reference point, it cannot replace real ATM assessment if the real-life performance of the client needs to be known. In terms of implications for future research, sufficient compelling evidence exists to encourage further randomized controlled trials. It might be useful if future studies of virtual reality training could be repeated with larger sample sizes, and if the treatment effect over time could be measured through follow-up studies.

## Conclusion

We found the VR-ATM to be a valid assessment and training tool for relearning the use of ATMs in clients with ABI. The VR-ATM, which can be openly accessed anywhere through the internet, provides information about the time and levels of cues needed in virtual practice, which is important for training skills in a protective environment, increasing the confidence of clients, and providing training opportunities prior to real-life practice in the community.

## Competing interests

The authors declare that they have no competing interests.

## Authors' contributions

KNKF designed the VR-ATM. CKKY formulated concepts and ideas in Part I of the study. WAKY and YEWH collected and analyzed data for Part I. KNKF formulated concepts in Part II of the study. CBCH, LKCK, LJCK, and LTHY collected and analyzed data for Part II. KNKF, CKKY and WAKY drafted the manuscript. All authors read and approved the final manuscript.

## Supplementary Material

Additional file 1 Behavioral Checklist in ATM operation. Click here for file

## References

[B1] Kobayashi I Iwazaki A Sasaki K FACT-V: Universal access and quality of interaction for Automatic Teller Machine (ATM) Interactive poster, Japan: 6th ERCIM Workshop 2006 "User Interfaces for All"; Japan 2006

[B2] Akatsu H Miki H Usability research for the elderly people Oki Technical Review (Special Issue on Human Friendly Technologies) 2004 71 3 54 57

[B3] Giles MG Clark-Wilson J Brain injury rehabilitation: A neurofunctional approach 1993 London: Chapman & Hall 10.3109/02699052.2014.946449 PMC4266071 25153760

[B4] Broeren J Claesson L Goude D Rydmark M Sunnerhagen KS Virtual rehabilitation in an activity centre for community-dwelling persons with stroke: The possibilities of 3-dimensional computer games Cerebrovasc Dis 2008 26 289 296 10.1159/000149576 18667809

[B5] Henderson A Korner-Bitensky N Levin M Virtual reality in stroke rehabilitation: A systematic review of its effectiveness for upper limb motor recovery Top Stroke Rehabil 2007 14 2 52 61 10.1310/tsr1402-52 17517575

[B6] Crosbie JH Lennon S Basford JR McDonough SM Virtual reality in stroke rehabilitation: Still more virtual than real Disabil Rehabil 2007 29 14 1139 1146 10.1080/09638280600960909 17613000

[B7] Christiansen C Abreu B Ottenbacher K Huffman K Masel B Culpepper R Task performance in virtual environments used for cognitive rehabilitation after traumatic brain injury Arch Phys Med Rehabil 1998 79 888 892 10.1016/S0003-9993(98)90083-1 9710158

[B8] Grealy MA Johnson DA Rushton SK Improving cognitive function after brain injury: The use of exercise and virtual reality Arch Phys Med Rehabil 1999 80 661 667 10.1016/S0003-9993(99)90169-7 10378492

[B9] Davies RC Lofgren E Wallergard M Linden A Boschian K Minor U Sonesson B Johansson G Three applications of virtual reality for brain injury rehabilitation of daily tasks Proceedings of the 4th International Conference on Disability, Virtual Reality & Associated Technology 2002: Sept, 2002 2002 Hungary; Vesaprem 93 100

[B10] Rizzo AA Bowerly T Buckwalter JG Schultheis M Matheis R Shahabi C Neumann U Kim L Sharifzadeh M Virtual environments for the assessment of attention and memory processes: The virtual classroom and office Proceedings of the International Conference on Disability, Virtual Reality & Associated Technology 2002: Sept, 2002 2002 Hungary: Vesaprem 17 19

[B11] Zhang L Abreu BC Seale GS Masel B Christiansen CH Ottenbacher KJ A virtual reality environment for evaluation of a daily living skill in brain injury rehabilitation: Reliability and validity Arch Phys Med Rehabil 2002 84 1118 1124 10.1016/S0003-9993(03)00203-X 12917848

[B12] Chow K Wong A Yan E Fong K Effectiveness of the virtual reality assessment and training in automatic teller machine (ATM) for clients with acquired brain injury (ABI) Paper presented at the Hospital Authority Kowloon Central Cluster (KCC) Convention: 28 April 2008; Hong Kong 2008

[B13] Fong KNK Howie D The effects of an explicit problem-solving skills training program using a metacomponential approach for outpatients with acquired brain injury Am J Occupational Therapy 2009 63 5 525 534 10.5014/ajot.63.5.525 19785251

[B14] Chiu HFK Lee HC Chung WS Kwong PK Reliability and validity of the Cantonese version of Mini-Mental State Examination: A preliminary study J Hong Kong College of Psychiatry 2004 4 25 28

[B15] Brain Injury Association of America Facts about traumatic brain injury http://www.biausa.org/aboutbi.htm

[B16] Niemiec RP Walberg HJ Comparative effects of computer-assisted instruction: A synthesis of reviews J Edu Computing Res 1987 3 1 19 37

[B17] Lau A Luk C Chan C Fung E Fong K Wong R Yui S Guidebook on Cognitive Training for Occupational Therapists 2002 Hong Kong: Hong Kong Occupational Therapy Association

[B18] Chan CCH Lee TMC Fong KNK Lee C Wong V Cognitive profile for Chinese patients with stroke Brain Inj 2002 16 10 873 884 10.1080/02699050210131975 12419000

[B19] NCNG, Inc Manual for the Neurobehavioral Cognitive Status Examination 1998 Fairfax: Northern California Neurobehavioral Group, Inc.

[B20] Portney LG Watkins MP Foundations of clinical research: Applications to practice 1993 Connecticut: Appleton & Lange 79 81

[B21] Fong KNK Chan MKL Ng PPK Ng SSW Measuring processing speed after traumatic brain injury in the outpatient clinic Neurorehabil 2009 24 2 165 173 10.3233/NRE-2009-0465 19339755

