# Supplementary material for: Usability of a virtual reality environment simulating an automated teller machine for assessing and training persons with acquired brain injury
Source: J Neuroeng Rehabil. 2010 Apr 30;7:19. doi: 10.1186/1743-0003-7-19 (PMC2881048; doi:10.1186/1743-0003-7-19)
Supplement: Additional file 1 — Behavioral Checklist in ATM operation. [file 1743-0003-7-19-S1.DOCX]

**Additional file 1 - Behavioral Checklist in ATM operation**

Please put (1) or (0) as appropriate, and choose (A) for cash withdrawals or (B) for money transfers:

| Basic operation | Pass (1) | Fail (0) | Comment |
| --- | --- | --- | --- |
| 1. Able to recognize the ATM machine |  |  |  |
| 1. Able to recognize the ATM card from wallet |  |  |  |
| 1. Able to recognize the ATM card slot in machine |  |  |  |
| 1. Able to put the ATM card in correctly |  |  |  |
| 1. Able to recognize the numbers on ATM panel |  |  |  |
| 1. Able to enter the password correctly |  |  |  |
| 1. Able to advance the process by pressing ‘enter’ |  |  |  |
| 1. Able to recognize the services key on ATM panel |  |  |  |
|  |  |  |  |
| (A) Cash withdrawals | Pass (1) | Fail (0) | Comment |
| 1. Able to choose cash withdrawal service |  |  |  |
| 1. Able to enter correct amount of cash to be withdrawn |  |  |  |
| 1. Able to advance the process by pressing ‘enter’ |  |  |  |
| 1. Able to collect the ATM receipt |  |  |  |
| 1. Able to collect the ATM card |  |  |  |
| 1. Able to collect cash from ATM |  |  |  |
| Overall |  |  |  |
| (B) Money transfers | Pass (1) | Fail (0) | Comment |
| 9. Able to choose money transfer service |  |  |  |
| 10. Able to choose the right account for transferring cash |  |  |  |
| 11. Able to enter the right account number for accepting  cash |  |  |  |
| 12. Able to advance the process by pressing ‘enter’ |  |  |  |
| 13. Able to enter correct amount of cash to be transferred |  |  |  |
| 14. Able to advance the process by pressing ‘enter’ |  |  |  |
| 15. Able to choose ‘end the service’ button |  |  |  |
| 16. Able to collect the ATM receipt |  |  |  |
| 17. Able to collect the ATM card |  |  |  |
| Overall |  |  |  |
